# Unique Spatial Immune Profiling in Pancreatic Ductal Adenocarcinoma with Enrichment of Exhausted and Senescent T Cells and Diffused CD47-SIRPα Expression

**DOI:** 10.3390/cancers12071825

**Published:** 2020-07-07

**Authors:** Alexandros Papalampros, Michail Vailas, Konstantinos Ntostoglou, Maria Lopez Chiloeches, Stratigoula Sakellariou, Niki V. Chouliari, Menelaos G. Samaras, Paraskevi D. Veltsista, Sofia D. P. Theodorou, Aggelos T. Margetis, Anna Bergonzini, Lysandros Karydakis, Natasha Hasemaki, Sophia Havaki, Ioannis I. Moustakas, Antonios Chatzigeorgiou, Timokratis Karamitros, Eleni Patsea, Christos Kittas, Andreas C. Lazaris, Evangelos Felekouras, Vassilis G. Gorgoulis, Teresa Frisan, Ioannis S. Pateras

**Affiliations:** 1 First Department of Surgery, Laikon University Hospital, National and Kapodistrian University of Athens, 11527 Athens, Greece; apapalampros@med.uoa.gr (A.P.); mike_vailas@yahoo.com (M.V.); lys_karydakis@yahoo.gr (L.K.); natashahashem@live.com (N.H.); felek@med.uoa.gr (E.F.); 2 Molecular Carcinogenesis Group, Department of Histology and Embryology, School of Medicine, National and Kapodistrian University of Athens, 11527 Athens, Greece; kostasntostoglou@gmail.com (K.N.); nikki.chouliari.94@gmail.com (N.V.C.); menelaos.g.samaras@gmail.com (M.G.S.); p.veltsista@student.vu.nl (P.D.V.); sofiatheo8@gmail.com (S.D.P.T.); margetis.aggelos.md@gmail.com (A.T.M.); shavaki@med.uoa.gr (S.H.); ckittas@med.uoa.gr (C.K.); vgorg@med.uoa.gr (V.G.G.); 3 Department of Molecular Biology, Umeå University, 90187 Umeå, Sweden; maria.chiloeches@umu.se (M.L.C.); anna.bergonzini@umu.se (A.B.); teresa.frisan@umu.se (T.F.); 4 First Department of Pathology, School of Medicine, National and Kapodistrian University of Athens, 11527 Athens, Greece; sakellarioustrat@yahoo.gr (S.S.); alazaris@med.uoa.gr (A.C.L.); 5 Oncology, School of Medical Sciences, Vrije Universiteit Amsterdam, De Boelelaan, 1117 Amsterdam, The Netherlands; 6 Department of Physiology, Medical School, National and Kapodistrian University of Athens, 11527 Athens, Greece; johnmoustakas86@gmail.com (I.I.M.); achatzig@med.uoa.gr (A.C.); 7 Institute for Clinical Chemistry and Laboratory Medicine, University Clinic Carl Gustav Carus, Technische Universität Dresden, 01307 Dresden, Germany; 8 Bioinformatics and Applied Genomics Unit, Department of Microbiology, Hellenic Pasteur Institute, 11521 Athens, Greece; tkaram@pasteur.gr; 9 Department of Pathology, Metropolitan General Hospital of Athens, 15562 Cholargos, Greece; elenipats@gmail.com; 10 Faculty of Biology, Medicine and Health Manchester Cancer Research Centre, Manchester Academic Health Sciences Centre, The University of Manchester, Manchester M20-4GJ, UK; 11 Center for New Biotechnologies and Precision Medicine, Medical School, National and Kapodistrian University of Athens, 75 Mikras Asias Str, 11527 Athens, Greece; 12 Basic Research, Biomedical Research Foundation of the Academy of Athens, 11527 Athens, Greece; 13 Department of Cell and Molecular Biology, Karolinska Institutet, 17177 Stockholm, Sweden

**Keywords:** pancreatic ductal adenocarcinoma, spatial heterogeneity, draining lymph nodes, tumor microenvironment, T cell exhaustion, T cell senescence, neoadjuvant chemotherapy, macrophage checkpoint, CD47, signal regulatory protein alpha (SIRPα)

## Abstract

Background: Pancreatic ductal adenocarcinoma (PDAC) is resistant to single-agent immunotherapies. To understand the mechanisms leading to the poor response to this treatment, a better understanding of the PDAC immune landscape is required. The present work aims to study the immune profile in PDAC in relationship to spatial heterogeneity of the tissue microenvironment (TME) in intact tissues. Methods: Serial section and multiplex in situ analysis were performed in 42 PDAC samples to assess gene and protein expression at single-cell resolution in the: (a) tumor center (TC), (b) invasive front (IF), (c) normal parenchyma adjacent to the tumor, and (d) tumor positive and negative draining lymph nodes (LNs). Results: We observed: (a) enrichment of T cell subpopulations with exhausted and senescent phenotype in the TC, IF and tumor positive LNs; (b) a dominant type 2 immune response in the TME, which is more pronounced in the TC; (c) an emerging role of CD47-SIRPα axis; and (d) a similar immune cell topography independently of the neoadjuvant chemotherapy. Conclusion: This study reveals the existence of dysfunctional T lymphocytes with specific spatial distribution, thus opening a new dimension both conceptually and mechanistically in tumor-stroma interaction in PDAC with potential impact on the efficacy of immune-regulatory therapeutic modalities.

## 1. Introduction

Pancreatic ductal adenocarcinoma (PDAC) has a poor prognosis with a 5- and 10-year survival rate of less than 10% and 5%, respectively [1]. Despite advances in oncology there has been little progress in the early diagnosis, prognosis and treatment of patients with PDAC. Accumulating evidence has demonstrated distinct molecular subtypes in PDAC which open a window for new therapeutic approaches and enable the implementation of personalized treatment [2].

A significant percentage of PDAC patients exhibit marked immune cell infiltrate characterized by increased T lymphocyte infiltration. Interestingly, a subset of these patients exhibit up-regulation of two druggable negative immune checkpoint inhibitors, namely programmed death 1 (PD-1) and cytotoxic T lymphocyte antigen 4 (CTLA-4) [3,4]. Based on these data, selective blockade of the programmed death ligand 1 (PD-L1)/PD-1 axis should provide a survival benefit in PDAC. However, the results of single agent immunotherapy blocking the PD-1/PD-L1 axis as well as treatment with CTLA-4 antagonists were disappointing [5]. The absence of criteria for patient selection may provide an explanation for these findings. Alternatively, but not mutually exclusive, the lack of efficiency can be attributed to the highly immunosuppressive microenvironment in PDAC, which can be associated with an exhausted T cell phenotype. It is well-established that the PD-L1 and PD-1 interaction promotes T cell exhaustion that is characterized by poor effector capabilities of T cells [6]. Interestingly, increased PD-1 expression by CD8+ tumor infiltrating T lymphocytes has been observed in PDAC [7,8,9]. Together, these studies suggest that to overcome immune checkpoint blockade failure in PDAC, a better understanding of the spatial distribution of T cells along with their functional status is required. 

Cancer cells can also hijack innate immune checkpoints including the CD47–signal regulatory protein alpha (SIRPα) phagocytosis checkpoint. CD47 is a transmembrane protein that is expressed essentially by all normal cells [10]. SIRPα is an inhibitory receptor normally expressed on myeloid cells that recognizes CD47 as a ligand. Binding of CD47 to SIRPα suppresses phagocytosis. Accumulating evidence demonstrates that the CD47-SIRPα axis is up-regulated in various human solid and hematologic tumors [11]. However, to the best of our knowledge, spatial characterization of SIRPα-CD47 signaling in PDAC patients has not been addressed yet. 

Based on these findings, a more detailed in situ characterization of the pancreatic cancer microenvironment will provide critical insights into the causes of limited efficacy of immunotherapy. This work aims to dissect for the first time the immune landscape in PDAC at protein and gene expression levels with single-cell resolution in intact tissues in relationship to the spatial heterogeneity in the tumor microenvironment, employing serial section analysis and multiplex in situ assays from the primary tumor and draining lymph nodes, with or without metastatic lesions.

## 2. Results

### 2.1. Distinct Profile of T Lymphocytes in Tumor Center (TC) and Invasive Front (IF) in Primary Lesions 

**Figure 1:**
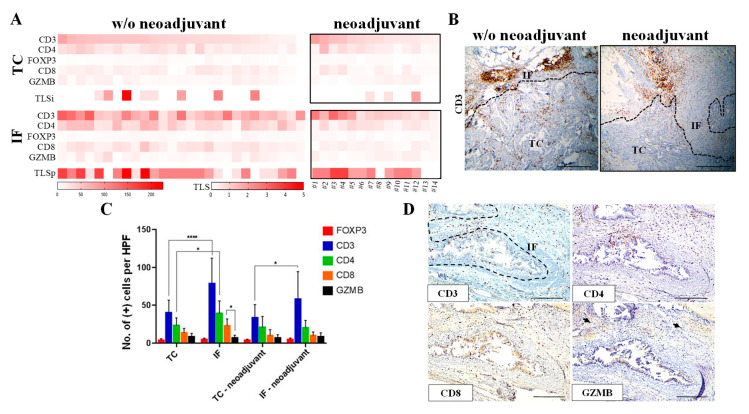
Distinct immune profile in the tumor center (TC) and the invasive front (IF) in pancreatic ductal adenocarcinoma (PDAC) patients. (**A**) Heatmap analysis of the protein expression of immune-related markers in the TC and the IF of PDAC receiving or not receiving neoadjuvant chemotherapy. Cases that received neoadjuvant chemotherapy are numbered in the heat map analysis from left to right as 1–14 (see Table S2). TLSi: tertiary lymphoid structures (intratumoral); TLSp: tertiary lymphoid structures (peritumoral). (**B**) Representative CD3 immunohistochemical staining showing increased CD3 positive cells (brown color) in the invasive front (IF) versus the tumor center (TC) in a patient without (w/o) neoadjuvant treatment (left panel) and a patient who received neoadjuvant treatment (right panel). Dashed line depicts the IF. Scale bar: 500 μm. (**C**) Quantification of the positive cells for indicated markers, assessed by immunohistochemistry. Data are reported as number of positive cells per high power field (HPF, magnification 400×). IF: invasive front; TC: tumor center. * *p*-value < 0.05, **** *p*-value < 0.0001. Data are expressed as mean ± standard deviation (STDEV) (N = 27, patients without neoadjuvant therapy; N = 14, patients with neoadjuvant chemotherapy). (**D**) Representative micrographs from serial section analysis assessing the expression of the T cell specific markers CD3, CD4, CD8, and granzyme B (GZMB) in primary lesions. Dashed line depicts the IF. Arrows denote positive GZMB immunostaining.

To elucidate the spatial differences of immune composition among patients who received or did not receive neoadjuvant chemotherapy, we analyzed the status of CD3, CD8, CD4, and FOXP3 in the tumor center (TC) and the invasive front (IF) in a panel of serial section from 42 PDACs (Table S1), 14 of which derived from patients who received neoadjuvant chemotherapy (Table S2). The immunohistochemical analysis revealed that the majority of PDAC patients exhibited significantly higher infiltration of total lymphocytes (CD3+) in the IF versus the TC (Figure 1A–C, *p*-value < 0.05), which is consistent with previous findings in PDAC [4,12,13]. A similar pattern for T cell expression was also observed in patients who received neoadjuvant chemotherapy (Figure 1A–C). The majority of CD3+ cells were CD4+ in agreement with a previous study [14] (Figure 1A,C,D). The prevalence of FOXP3-positive T regulatory cells exhibited no significant differences between the TC and the IF in both patient subgroups. Overall, we observed a distinct profile in subsets of T lymphocytes in the TC and IF that was similar both in chemotherapy-naïve and patients receiving neoadjuvant chemotherapy.

The immunohistochemical analysis was complemented with a morphological feature with a prognostic value, namely the presence of tertiary lymphoid structures (TLS) [15], which develop in peripheral tissues upon persistent exposure to inflammatory stimuli. Given that intratumoral TLS are associated with a favorable prognosis in PDAC [16], both intratumoral (TLSi) and peritumoral TLS (TLSp) were assessed. Presence of TLS was more prominent in the IF compared to that observed in the TC in both groups of PDAC patients (Figure 1A). 

To assess the cytolytic potential of T lymphocytes we evaluated the status of granzyme B (GZMB) in the TC and the IF. The percentage of cells positive for this marker was consistently lower than the percentage of CD8-positive cells, irrespective of the neoadjuvant treatment, suggesting that only a subset of CD8+ lymphocytes were cytotoxic (Figure 1A,C,D).

### 2.2. T Cells Infiltrating Pancreas Primary Cancer Microenvironment Exhibit Features of T Cell Exhaustion and Senescence

**Figure 2:**
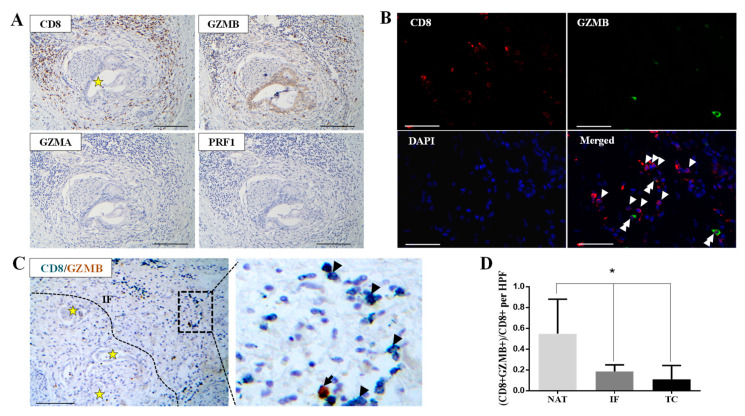
Decreased cytotoxic potential in CD8+ cells in PDAC. (**A**) Representative micrographs from serial section analysis demonstrating decreased granzyme A (GZMA), perforin 1 (PRF1), and granzyme B (GZMB) immunostaining in the IF in PDAC. Asterisk denotes perineural invasion by cancer glands. Scale bar: 100 μm. (**B**) Representative micrographs showing the expression of CD8 (red) and granzyme B (green) in the tumor center (TC). Nuclei were counterstained with 4′,6-diamidino-2-phenylindole (DAPI) (blue). Arrowheads indicate CD8+ cells, and double arrowheads indicate CD8+/GZMB+ cells. Scale bar: 100 μm. (**C**) Representative micrographs showing GZMB (brown) and CD8 (emerald) expression assessed by double immunohistochemical staining in the invasive front (IF) from the PDAC case presented in (A). Arrowheads indicate CD8+ cells, and the arrow indicates a CD8+/GZMB+ cell. Dashed line delineates the IF. Asterisks depict cancer glands. Scale bar: 200 μm. (**D**) Quantification of the percentage of GZMB/CD8 double positive cells in the invasive front (IF), the tumor center (TC), and the normal parenchyma adjacent to the tumor (NAT). * *p*-value < 0.05. Data are expressed as mean ± STDEV (N = 6).

Based on our initial observation that CD8-positive cells exhibit decreased GZMB expression (Figure 1A,C,D), we performed immunohistochemistry on serial sections and double in situ immunostaining on the same section in order to examine the expression of CD8 and three cytolytic effectors, granzyme A (GZMA), granzyme B (GZMB), and perforin 1 (PRF1) in the TC and the IF. CD8/GZMB/GZMA/PRF1 serial section analysis (Figure 2A and Figure S1A), CD8/GZMB double immunofluorescence (Figure 2B), and CD8/GZMB double immunohistochemistry (Figure 2C) revealed a lower percentage of cells positive for all three cytolytic effector proteins compared to the total number of CD8-positive T lymphocytes both in the TC and the IF. Interestingly, the fraction of CD8+/GZMB positive T lymphocytes appeared in significantly higher proportion in the normal pancreatic parenchyma adjacent to the tumor (NAT) than in the corresponding IF and TC (Figure 2D and Figure S1B,C; *p*-value < 0.05).

**Figure 3:**
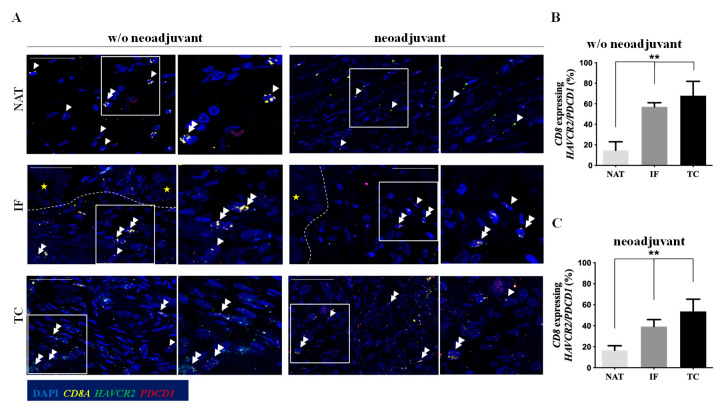
CD8+ T lymphocytes in the tumor center (TC) and the invasive front (IF) exhibit an exhausted phenotype. (**A**) RNAscope analysis to examine the co-expression of *HAVCR2* (green) and/or *PDCD1* (red) and *CD8A* (yellow) in PDAC patients. Representative confocal micrographs in PDAC patients without (w/o) neoadjuvant therapy and PDAC patients who received neoadjuvant chemotherapy. Dashed line delineates the invasive front (IF). Yellow asterisks depict cancer glands. Double arrowheads indicate *CD8A*-positive cells co-expressing *HAVCR2/PDCD1* and single arrowheads depict only *CD8A* expressing T lymphocytes. Scale bar: 100 μm (**B**) Quantification of CD8+ T lymphocytes expressing *HAVCR2/PDCD1* mRNA in PDAC patients who did not receive neoadjuvant therapy. ** *p*-value < 0.01. Data are expressed as mean ± STDEV (N = 5). (**C**) Quantification of CD8+ T lymphocytes expressing *HAVCR2/PDCD1* mRNA in PDAC patients who received neoadjuvant chemotherapy. ** *p*-value < 0.01. Data are expressed as mean ± STDEV (N = 3). Tumor center (TC), invasive front (IF), normal parenchyma adjacent to the tumor (NAT).

Loss of GZMB production alongside with up-regulation of a diverse arrays of inhibitory receptors (IRs) is a feature of CD8+ T lymphocytes exhibiting an advanced exhausted phenotype [6]. Therefore, using a multiplex RNAscope, we assessed the level of mRNA expression of *CD8A* as well as two common IRs up-regulated in T cells with an exhausted phenotype: *PDCD1* (alternatively known as PD-1), and *HAVCR2* (hepatitis A virus cellular receptor 2, also known as TIM3). Negative control probes and probes specific for three housekeeping genes with different expression levels were used to evaluate the RNA quality (Figure S2). Approximately 40% to 70% of *CD8A*+ T lymphocytes co-expressed *PDCD1* and/or *HAVCR2* in both IF and TC, independently of the neoadjuvant chemotherapy (Figure 3A–C), suggesting that T cytotoxic lymphocytes are exhausted in the pancreatic cancer microenvironment. In line with the CD8/GZMB data, the levels of *CD8A*+ cells co-expressing *PDCD1*/*HAVCR2* were significantly lower in the NAT (Figure 3B,C, *p*-value < 0.01).

We further assessed the presence of exhausted T cells in representative non-malignant pancreatic lesions. As shown in Figure S3, the percentage of *CD8A*-positive T lymphocytes expressing *HAVCR2* and/or *PDCD1* was lower in cases of intraductal papillary mucinous neoplasms (IPMN) and serous cystadenoma (SC) compared to the percentage observed in PDAC. Interestingly, in chronic pancreatitis the percentage of exhausted T cells was similar to that observed in the PDAC primary tumors (Figure 3 and Figure S3), possibly due to a diffuse and continuous inflammatory condition that potentially favors T cell exhaustion.

**Figure 4:**
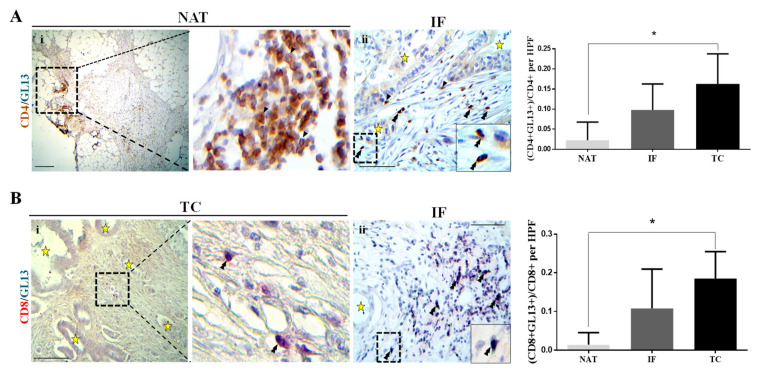
T lymphocytes in PDAC exhibit a senescent phenotype. (**A**) Co-expression of CD4 (brown) and GL13 (emerald) assessed by double immunohistochemistry analysis. Left panel: representative micrograph showing CD4 single positive (single arrows) in the normal parenchyma adjacent to the tumor (NAT) (**i**, scale bar: 200 μm). Insert indicates higher magnification. CD4 and GL13 double positive cells (double arrowheads) in the tumor center (TC) (**ii**, scale bar: 100 μm). Asterisks depict cancer glands. Right panel: quantification of CD4 and GL13 double positive cells per high power field (HPF, 400× magnification). * *p*-value < 0.05. Data are expressed as mean ± STDEV (N = 5). (**B**) Co-expression of CD8 (red) and GL13 (emerald) assessed by double immunohistochemistry analysis. Left panel: representative micrographs of the TC (**i**) and IF (**ii**), where double arrowheads depict CD8 and GL13 double positive cells. Asterisks denote cancer glands. Scale bar: 200 μm (i); 100 μm (ii). Insert depicts higher magnification. Right panel: quantification of the CD8 and GL13 double positive cells per high power field (HPF, 400× magnification). * *p*-value < 0.05. Data are expressed as mean ± STDEV (N = 5).

Prompted by a previous study demonstrating that senescent CD8+ T cells express decreased PRF1 and GZMB [17], we investigated whether T lymphocytes in the PDAC have acquired a senescent phenotype. To address this issue, a two-step in situ assay was performed to assess the level of expression of the surface T cell markers CD4 and CD8 by immunohistochemistry, followed by a hybrid histo-/immunochemical assay employing GL13 (SenTraGor^TM^). The analysis demonstrated increased levels of cells double positive for CD4/GL13 and CD8/GL13 in the pancreatic cancer microenvironment that reached a statistical significance in the TC versus NAT (Figure 4A,B, *p*-value < 0.05).

### 2.3. T Cells in Metastatic Lymph Nodes Exhibit Features of T Cell Exhaustion and Senescence

**Figure 5:**
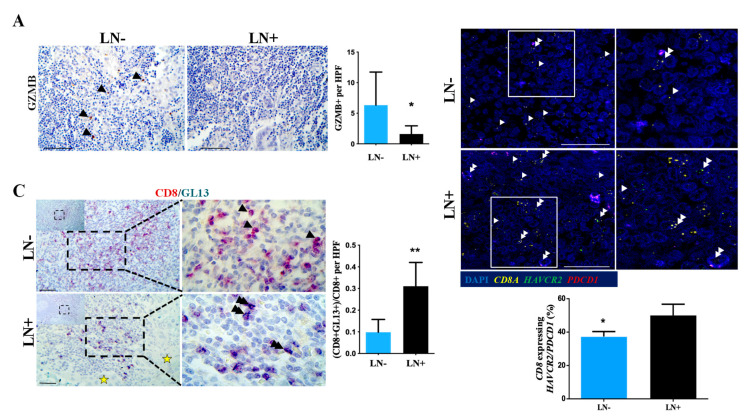
T cells in positive draining lymph nodes exhibit features of T cell exhaustion and senescence. (**A**) Left panel: representative micrograph of GZMB immunohistochemical staining (brown) of non-metastatic (LN-) or metastatic lymph nodes (LN+) from the same patient. Scale bar: 100 μm. Right panel: quantification of GZMB-positive cells. * *p*-value < 0.05. Data are expressed as mean ± STDEV (N = 9). (**B**) RNA scope analysis to examine the mRNA expression levels of *HAVCR2* (green), *PDCD1* (red), and *CD8A* (yellow) in LN+ and LN-. Upper panel: representative confocal micrographs. Double arrowheads depict *CD8A*+ co-expressing *HAVCR2/PDCD1* and single arrowheads demonstrate *CD8A*+ single positive T lymphocytes. Scale bar: 100 μm. Lower panel: quantification of CD8+ T lymphocytes expressing *HAVCR2/PDCD1* mRNA. * *p*-value < 0.05. Data are expressed as mean ± STDEV (N = 6). (**C**) Co-expression of CD8 (red) and GL13 (emerald) assessed by double immunohistochemistry analysis in non-metastatic (LN-) or metastatic lymph nodes (LN+) from the same patient. Left panel: representative micrograph where double arrowheads depict CD8 and GL13 double positive cells and single arrowheads indicate CD8 single positive T lymphocytes. Scale bar: 100 μm. Asterisk depicts cancer glands in the metastatic lymph node (LN+). Right panel: quantification of the CD8 and GL13 double positive cells per high power field (HPF, 400× magnification). ** *p*-value < 0.01. Data are expressed as mean ± STDEV (N = 9).

We next assessed the functional status of T lymphocytes in matched tumor negative and positive regional lymph nodes from PDAC patients. The presence of CD8-positive T cells exhibiting the features of exhaustion was higher in the regional metastatic lymph nodes compared to non-metastatic lymph nodes, as assessed by the lower levels of GZMB+ cells (Figure 5A, *p*-value < 0.05) along with an increased proportion of *CD8A* T cells co-expressing *PDCD1* and/or *HAVCR2* (Figure 5B, *p*-value < 0.05). Similarly, the levels of CD8+/GL13+ T lymphocytes were significantly elevated in lymph nodes with metastatic lesions compared with negative lymph nodes (Figure 5C, *p*-value < 0.01). Taken together this analysis clearly demonstrates the co-existence of exhausted and senescent T cell subpopulations in pancreatic primary cancer microenvironment and metastatic lymph nodes.

### 2.4. Immune Response 2 Predominates in PDAC and Is More Pronounced in the TC

Macrophages constitute a dominant population in pancreatic cancer microenvironment [5]. Therefore, we examined the status of CD64 (FcγRI) and CD206 (mannose receptor) which are expressed by macrophages with a classical (M1) and alternatively activated (M2) phenotype, respectively [18,19]. A significant percentage of both intratumoral (TC) and peritumoral (IF) macrophages exhibited an M2-like phenotype, demonstrated by the higher CD206 versus CD64 immunopositivity (Figure S4A–C, *p* < 0.05). This conclusion was confirmed by the higher percentage of cells expressing CD163, an additional marker for alternatively activated macrophages (Figure S4C). The prevalence of a higher percentage of CD206 and CD163 positive cells was independent of the neoadjuvant chemotherapy treatment, and was specific for the TME, since cases with non-cancerous pancreatic lesions expressed diffused CD64, CD163, and CD206 immunopositivity (Figure S4C,D), in accordance with a previous study showing high CD204+ and CD163+ staining in non-cancerous pancreatic lesions [12].

Since alternatively activated macrophages are related with a type 2 immune response [20], we next investigated by multiple in situ RNAscope the expression levels of two T cell transcription factors, which are characteristic of the inflammatory type 1 or immunoregulatory type 2 adaptive immune responses [21]: *TBX21* (also known as *T-bet*) and *GATA3* (*GATA-binding protein 3*), respectively. We observed that *GATA3/TBX21* ratio is significantly increased in TC compared to IF and NAT (Figure S5A,B, *p*-value < 0.05). Additionally, prompted by a previous study demonstrating that interleukin 13 (IL13) favors the accumulation of alternative activated macrophages during pancreatic carcinogenesis [22], we examined the status of *IL13*. The assessment of the mRNA expression levels of *IL13*, revealed a higher *IL13/TBX21* ratio in the TC compared to that observed in the IF and NAT (Figure S5C) similar to that of *GATA3/TBX21*. Overall, these findings demonstrate that in PDAC the local microenvironment is directed towards a type 2 immune response, which is more prominent in the TC compared to the IF.

### 2.5. An Emerging Role of Macrophage Checkpoint in PDAC

The status of PD-L1/PD-1 and CD47-SIRPα, two immune-related druggable axes, was further examined.

**Figure 6:**
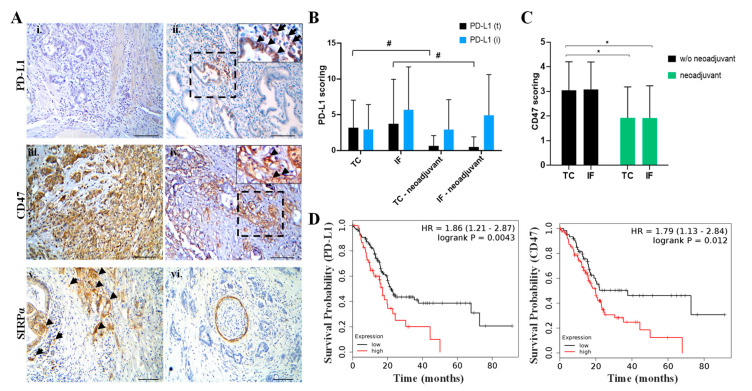
Status of programmed death ligand 1/programmed death 1 (PD-L1/PD-1) and CD47–signal regulatory protein alpha (SIRPα) axes and their clinical relevance in PDAC. (**A**) **i**–**vi**. Expression of PD-L1, CD47, and SIRPα assessed by immunohistochemistry analysis. **i.** Representative case with negative PD-L1 expression in the invasive front (IF). **ii.** PD-L1 expression in tumor cells and mononuclear inflammatory cells in the IF (**ii**). Diffused CD47 expression in the IF (**iii**) and the tumor center (TC) (**iv**). Inserts denote areas with positive CD47 immunostaining. **v.** Expression of SIRPα in the IF both in cancer glands (arrowheads) and in the surrounding stroma (arrows). **vi.** Cross section of peripheral nerve expressing SIRPa, which serves as an internal positive control. Scale bar: 100 μm. (**B**) Quantification of PD-L1 scoring. # strong tendency. Data are expressed as mean ± STDEV (N = 27, without neoadjuvant therapy; N = 14, received neoadjuvant chemotherapy). (**C**) Quantification of CD47 scoring. * *p*-value < 0.05. Data are expressed as mean ± STDEV (N = 27, without neoadjuvant therapy; N = 14, with neoadjuvant chemotherapy). (**D**) Kaplan–Meier survival curve on PD-L1 and CD47 mRNA status in PDAC demonstrates that elevated *PD-L1* and *CD47* expression is associated with poor survival (N = 177).

Expression of PD-L1 in the tumor cells (PD-L1(t)) and the surrounding immune cells (PD-L1(i)) was assessed employing two PD-L1 specific antibodies: clone SP263 and clone 28-8. The majority of PDAC cases exhibited restricted PD-L1 expression both in the TC and the IF (Figure 6Ai,ii and Figure S4A), with a high concordance between the two clones, although clone 28-8 staining revealed more tumor cell immunopositivity than the SP263. Interestingly, patients who received neoadjuvant chemotherapy exhibited a strong tendency of decreased PD-L1 expression on tumor cells both in the TC and the IF compared to non-treated patients (Figure 6B). PD-1 expression was restricted to stromal cells; the expression status was similar in chemotherapy-naïve and patients receiving neoadjuvant chemotherapy (Figures S4A and S6).

Evaluation of CD47-SIRPα axis revealed: a) diffuse CD47 expression in the majority of cases both in the TC and the IF (Figure 6Aiii–iv and Figure S4A), and similarly to the expression of PD-L1, the levels of CD47 were lower in patients who received neoadjuvant therapy (Figure 6C and Figure S7A, *p*-value < 0.05); b) SIRPα was detected both in stromal and cancer cells in the majority of PDAC cases, independently of the neoadjuvant chemotherapy (Figure 6v,vi and Figure S4A).

Previous data reported that the expression of PD-L1 or PD-1 in tumor-associated macrophages (TAMs) correlates with an attenuated phagocytic capacity [23,24]. Therefore, we investigated whether additional inhibitory checkpoint markers are expressed on macrophages present in the PDAC stroma. The analysis revealed the co-expression of PD-L1 and PD-1 with CD163 and CD206 positive macrophages, respectively (Figure S7B,C) in PDAC patients. These findings suggest that multiple mechanisms limit phagocytosis in PDAC.

The clinical significance of PD-L1 and CD47 expression was evaluated by bioinformatics analysis, integrating RNA-seq data from TCGA repository (including N = 177 patients with PDAC) and employing Kaplan–Meier plotter [25]. The data revealed that high levels of PD-L1 and CD47 status are significantly associated with a worse prognosis.

## 3. Discussion

Knowledge of the immunological landscape in PDAC has increased immensely. However, most of the data have been largely derived from a type of analysis in which tissue preservation is lost, with the following limitations: (a) unfeasible to study the immune profile at a single-cell level and the extent of stromal heterogeneity; (b) genes can be expressed in opposing directions in various cell types, thereby the average RNA values may appear unchanged; (c) gene expression of rare cell types may be missed. In addition, several studies have examined the immune composition performing in situ assays without taking into consideration spatial cancer heterogeneity. Here we provide novel insights into the pancreatic cancer immune landscape by taking into consideration spatial heterogeneity that allows several important and novel conclusions to be drawn on the pancreatic cancer microenvironment.

Sections analysis demonstrated a similar spatial distribution of T cytotoxic, T helper, and T regulatory cells in PDACs irrespectively of the administration of neoadjuvant chemotherapy. The majority of CD3+ cells were CD4+, and to a lesser extent CD8+, both in the TC and the IF, which agrees with a previous study [14]. FOXP3+ T regulatory cells accounted for a small percentage of CD3+ cells. A previous study demonstrated that neoadjuvant therapy correlated with reduced FOXP3+ cells in a cohort of 21 PDAC patients [9]. This discrepancy is likely attributed to the fact that the study by Shibuye et al. (2014) [9] included a mixed population of PDAC patients that received chemoradiotherapy or chemotherapy alone. Considering that a neoadjuvant therapy is often used, it is important to examine large cohorts of patients treated with different neoadjuvant regimens in order to study the impact of different pre-operative treatments on the immune landscape.

Notably, our analysis clearly demonstrates that dense desmoplastic stroma does not impede lymphocyte infiltration, in line with previous studies depicting a high T cell tumor infiltration in a significant percentage of PDAC patients, which is associated with a favorable prognosis [4,8,13,14,26]. Interestingly, one of these studies demonstrated that the presence of T cytotoxic cells in the direct vicinity to cytokeratin 8 positive cancer cells is correlated with a better prognosis [14]. The latter study further stressed the relevance of in situ analysis to define the spatial distribution of the immune cells.

Our data demonstrate that cytotoxic T cells infiltrating PDAC microenvironment exhibit features of T cell exhaustion, with reduced levels of expression of key cytolytic effectors (including GZMB, GZMA, PRF1) in the TC and the IF (Figure 2 and Figure S1), in agreement with bioinformatics analysis utilizing publicly available transcriptomic data that revealed reduced cytolytic T cell activity in PDAC [3,27]. Hence, the increased tumor-infiltrating T effector lymphocytes in PDAC tumors does not necessary mean that this T cell population is functional. Since T cell exhausted cells exhibit progressive loss of cytotoxicity [6], we demonstrated for the first time that CD8+ T lymphocytes in the TC and the IF exhibit an exhaustive phenotype irrespective of the administration of neoadjuvant chemotherapy, strongly suggesting that T cell exhaustion is a robust phenomenon in PDAC microenvironment.

Together our findings indicate that there is a direct correlation between the presence of tumor cells and the T exhausted phenotype, since the percentage of T cells with an exhausted phenotype was significantly lower in NAT and non-metastatic LNs compared to the TC, IF, and metastatic LNs from the same patients, excluding the confounding effect of different genetic backgrounds (Figure 3 and Figure 5A,B). The paracrine role of cancer cells in influencing the exhausted T cell phenotype is further supported by the lower percentage of these cell types in non-malignant pancreatic lesions, with the exception of the case of chronic pancreatitis (Figure S3). In the latter case, it is likely that the persistence of inflammatory stimuli could drive T cell exhaustion. The limited tissue availability from cases with chronic pancreatitis makes immune profiling difficult.

Persistent antigenic stimulation is a key mechanism driving T cell exhaustion. However, the limited T cell clonality in PDAC [8] suggests that other mechanisms are possibly driving T cell exhaustion in PDAC microenvironment. Soluble mediators including interleukin 10 (IL10), which is an immune response 2 derived anti-inflammatory cytokine, and transforming growing factor β (TGFβ) are abundant in PDAC microenvironment [5]. Both inhibitory cytokines can promote T cell exhaustion [28]. Additionally, and not mutually exclusive, CD4+ T regulatory cells foster T cell exhaustion through the secretion of IL10 and TGFβ [28].

A further characterization of the functionality of T lymphocytes demonstrated the presence of senescent T cells in PDAC microenvironment (Figure 4 and Figure 5C). Our analysis depicted for the first time that: a) a significant percentage of CD8+ and CD4+ lymphocytes exhibits a senescent phenotype in the TC, whereas in the NAT the percentage of these cells is significantly lower and b) the senescence phenotype correlates with metastatic tumor in lymph nodes. An essential question arising is what is the biological significance of T cells with a senescent phenotype in PDAC? Although evidence supports that T cell senescence is a dysfunctional state [28], the role of senescent T cells in the pancreatic cancer microenvironment is unknown. Do they act in a pro-tumorigenic or an anti-tumorigenic fashion? Our data open a new fascinating field of research dealing with the biology of different subpopulations of T cells in PDAC microenvironment.

Our data also demonstrate a higher CD206/CD64 expression ratio in PDAC microenvironment (Figure S4), which is considered as a surface marker for alternatively activated (M2) and classically activated (M1) macrophages, respectively [18,19]. Interestingly, alternatively activated macrophages secrete IL10 which in turn promotes T cell exhaustion, revealing a potential link between macrophages having a M2-like phenotype with T cell exhaustion in PDAC. In addition, alternatively activated macrophages are associated with a T helper 2 response [20]. Consistently, we found a predominant *GATA3* (T helper 2 marker) over *TBX21* (T helper 1 marker) status in the TC compared to the IF and the NAT, supporting the presence of type 2 immune response in the PDAC microenvironment, which was further corroborated by the detection of a higher percentage of cells expressing *IL13* mRNA (Figure S5), a direct target of GATA3 [29]. These data are in line with a previous study showing increased GATA3 versus T-bet immunostaining in PDAC, although the spatial distribution was not taken into consideration [30]. Furthermore, the decreased *TBX21* over *GATA3* status comes in line with a previous study showing a critical role of T-bet (the protein encoded by *TBX21*) in T cell exhaustion by directly down-regulating the inhibitory receptor PD-1 (encoded by *PDCD1*) [28]. Within this context down-regulation of T-bet may further augment PD-1 expression, a key IR expressed in T cells with an exhausted phenotype.

The analysis of the PD-L1/PD-1 axis demonstrated the reduced PD-L1 expression in the TC and the IF in PDAC relative to other malignancies [31]. Notably, the PD-L1 status was further decreased in patients treated with neoadjuvant chemotherapy (Figure 6B). At this point, it is important to underline that there are differences in PD-L1 staining pattern (in the tumor cells and the immune cells) among the available PD-L1 antibody clones. Discrepancies in the immunohistochemical detection of PD-L1 have been attributed to the different binding features of each clone [32], underscoring the importance to employ different clones to study PD-L1 status in PDAC patients. In our study we employed SP263 and 28-8 clones which are both validated to examine PD-L1 staining in human tumor samples. Our work correlates with previous studies employing E1L3N clone to study PD-L1 in PDAC patients although the topographical area was not taken into consideration [4,8,26,33,34,35]. These data suggest that anti-PD-L1/PD-1 monotherapy may have a limited efficacy in PDAC patients. Conversely, the combined PD-L1 and PD-1 expression on the same patients provides a rationale for blocking both PD-L1 and PD-1 in PDAC. Along this line, a recent study demonstrated that a combined PD-L1 and PD-1 blockade is advantageous compared to a monotherapy approach promoting tumor regression in an animal PDAC mouse model [36]. However, it remains to be clarified whether treatment with immune checkpoint inhibitors reinvigorate pre-existing T exhausted cells in PDAC microenvironment. Two recent studies in patients with different types of cancer challenge the conventional wisdom that anti-PD-1 and anti-PD-L1 therapy reboots existing intratumoral T cells with an exhausted phenotype [37,38]. Furthermore, the observation that high PD-L1 mRNA status is related with worse prognosis in PDAC as shown in the current study (Figure 6D), in agreement with a previous report [39], indicates that PD-L1 expression could serve as a prognostic marker.

We have also shown the expression of PD-1 and PD-L1 in the macrophage population, in line with previously reported data [8]. It is noteworthy that the PD-1/PD-L1 axis can also regulate phagocytosis in tissue associated macrophages (TAMs) [23,24], underscoring the potential promising effect of combined anti-PD-1 with anti-PD-L1 treatment in a subset of PDAC patients.

Our study further revealed that the expression of CD47 in the TC and the IF was significantly lower in patients treated with neoadjuvant chemotherapy (Figure 6C and Figure S7A). This finding becomes even more interesting given that high *CD47* expression predicts worse prognosis for PDAC patients (Figure 6D). Together, these data suggest the potential therapeutic application of CD47-SIRPα immune checkpoint blockade in PDAC patients. Indeed, pre-clinical studies demonstrated a promising effect of CD47 inhibition alone or in combination with gemcitabine in PDAC patient-derived xenograft settings [40,41]. Interestingly, the results from a first-in-human first-in-class phase I clinical trial blocking CD47 in advanced malignancies including four patients with PDAC demonstrated its favorable toxicity profile, opening a new dimension in immunotherapy [42].

## 4. Material and Methods

### 4.1. Clinical Samples

The collection of the human samples and their experimental use were approved by the Bioethics Committee of the Medical School of National and Kapodistrian University of Athens (Νο. 1617031060), in accordance with the Declaration of Helsinki and local laws and regulations, following written patient consent. The analysis included 42 patients with PDAC along with 9 patients with non-malignant pancreatic lesions including 2 patients with intraductal papillary mucinous neoplasm (IPMN), 2 patients with mucinous cystic neoplasm (MCN), 1 patient with serous cystadenoma (SC), 1 patient with pseudopapillary neoplasm (PSN), 2 patients with chronic pancreatitis (ChP), and 1 patient with main pancreatic duct lithiasis (mPDL). The histopathological evaluation was performed according to 2019 World Health Organization (WHO) classification of tumors of the digestive system. For PDAC cases, there were available sections including primary lesions harboring the invasive front (IF) and the tumor center (TC) along with matched draining lymph nodes (LNs) with and without metastatic lesions. A total of 14 patients received neoadjuvant chemotherapy. Clinicopathological data of all patients are summarized in Table S1. The treatment regimen of the 14 patients that received neoadjuvant chemotherapy is provided in Table S2.

### 4.2. Immunohistochemistry (IHC) and Immunofluorescence (IF)

IHC and IF were performed on formalin-fixed paraffin embedded (FFPE) tissues. The following antibodies were used: anti-CD3 (A0452, DAKO, 1:100); anti-CD4 (ab133616, Abcam, Cambridge, UK, 1:200); anti-CD8 (108M, Cell Marque, 1:60); anti-CD64 (ab140779, Abcam, 1:200); anti-CD206 (ab64693, Abcam, 1:1500); anti-CD163 (NCL-CD163, Novocastra-Leica, 1:100); anti-CD47 (ab226837, Abcam, 1:100); anti-SIRPα (ab53721, Abcam, 1:200); anti-FOXP3 (ab22510, Abcam, 1:100); anti-Granzyme A (ab10870, Abcam, 1:100); anti-Granzyme B (ab4059, Abcam, 1:300); anti-Perforin (ab75573, Abcam, ready to use); anti-PD-1 (ab52587, Abcam, 1:80). The PD-L1 staining was performed using: a) anti-PD-L1 (clone 28-8, ab205921, Abcam, 1:400) and b) the Ventana PD-L1 (clone SP263, Roche, Greece) assay. Antibodies were also sourced using BenchSci (www.benchsci.com). For single IHC analysis the UltraVision^TM^ Quanto Detection System horseradish peroxidase activity based on the activity of 3,3’-diaminobenzidine (HRP DAB) was used (#TL-125-QHD, Thermo Scientific, Greece) according to the manufacturer’s instructions. Regarding double IHC analysis the TripleStain IHC Kit (M&M&R on Human tissue DAB, AP/Red, and HRP/Green, ab183286 Abcam) was employed following the manufacturer’s instructions. For IHC and IF, antigen retrieval was heat-mediated in 10 mM citric acid (pH 6.0). Goat anti-mouse (Alexa Fluor^®^ 568, ab175473, Abcam) and goat anti-rabbit (Alexa Fluor^®^ 488, ab150077, Abcam) were used as secondary antibodies for double IF. Hematoxylin and DAPI were employed as counterstaining for IHC and IF, respectively. For CD3, CD8, CD4, FOXP3, CD206, CD64, GZMA, GZMB, PRF1, and PD-1 we counted the number of positive cells in the stroma per high power field (HPF, magnification 400×). CD47 scoring was based on Gu et al. (2018) [43] using a mixed score ((intensity index: 0–3) × (labeling index: 0–4)). For SIRPα we evaluated the percentage of staining in stromal and cancer cells, scored as 0 (0–1%), 1 (2–10%), 2 (11–25%), 3 (26–50%), or 4 (51–100%). For PD-L1 assessment we assessed separately the percentage of tumor viable cells (PD-L1(t)) and immune cells (PD-L1(i)) [44]. The maximum score was defined as 100. For the evaluation of double stainings we counted the following ratios per HPF: (a) (CD8+GZMB+)/(CD8+); (b) (GL13+CD8+)/(CD8+), and (c) (GL13+CD4+)/(CD4+). Human spleen served as a positive control for CD3, CD4, CD8, GZMA, GZMB, and PRF1. Human tonsil was used as positive control for FOXP3, PD-1, and PD-L1. Human liver served as positive control for CD206 and CD64. For SIRPα and for CD47, peripheral nerves and cases with prostate adenocarcinoma served as positive controls, respectively. A negative control was employed by omitting the primary antibody. To exclude false-positive immunofluorescence from the secondary antibodies used in double IF, we performed a parallel staining omitting each time one of the primary antibodies. Slide examination was performed by three independent observers with minimal inter-observer variability.

### 4.3. Evaluation of TLS

The detection and assessment of TLS were based on hematoxylin and eosin (H&E) staining. TLS were defined as dense cellular aggregates reminiscent of germinal centers or lymphocytic aggregates or lymphoid follicles devoid of germinal centers [15]. Specifically, for TLS evaluation, we counted the number of peritumoral and intratumoral TLS per 100× magnification.

### 4.4. Senescence

For the detection of senescent cells, we performed a hybrid histo-/immunochemical assay utilizing GL13, a lipophilic, biotin-linked Sudan Black-B (SBB) analogue (commercially available as SenTraGor^®^) [45]. GL13 binds to a non-degradable aggregate of oxidized molecules accumulating in the cytoplasm of senescent cells. Previously characterized cases served as positive controls [46].

### 4.5. RNA Scope

*In situ* detection of mRNA status for *HAVCR2*, *PDCD1*, *CD8A*, *IL13*, *TBX21*, and *GATA3* utilizing the RNAscope Multiplex Fluorescent Reagent Kit (Bio-techne, Oxford, UK) was performed according to manufacturer’s protocol. As positive control the housekeeping genes polymerase II subunit A (*Pol2a*), peptidylprolyl isomerase B (*PPIB*), and ubiquitin C (*UBC*) were used as internal controls to assess the mRNA quality. The particular cocktail of probes detect RNA with different expression levels: UBC: medium/high (more than 20 copies per cells); PPIB medium (10–30 copies per cells), and POLR2A low (3–15). A negative control probe was also employed to verify the absence of background. The sections were pretreated using Protease Plus for 30 min at 40 °C and further incubated with the probes targeting diluted according to manufacturer´s protocol for 2 h at 40 °C in HybEZ^TM^ Oven. For detection of the targeting probes, the sections were incubated with fluorescent dyes Opal 520, Opal 570, and Opal 690 (Akoya Biosciences, Sweden). The slides were washed using the supplied washing buffer (Bio-techne, Oxford, UK) after each hybridization step at room temperature. Nuclei were counterstained with DAPI. Images were acquired at BICU facilities at Umeå University using a confocal scanning microscope (Leica TCS SP8, Leica Microsystems, Wetzlar, Germany). To assess the percentage of positive *CD8A* cells co-expressing *HAVCR2* and/or *PDCD1*, we visually scored the number of *CD8A* cells with ≥1 dot of *HAVCR2* and/or *PDCD1* as follows: (*CD8A*-positive cells expressing *HAVCR2* and/or *PDCD1*)/(total number of *CD8A*-positive cells). To evaluate the ratio of *IL13/TBX21* and *GATA3/TBX21*, first we assessed quantitatively employing H-score, following manufacturer´s guidelines and then we divided the corresponding H-scores. For RNAscope evaluation in the primary lesions we examined separately three representative areas for each tissue area (the invasive front (IF), the tumor center (TC), and the normal pancreatic parenchyma adjacent to the tumor (NAT)). Similarly, the positive and the negative lymph nodes (LNs) and the non-malignant pancreatic lesions were examined by investigating three different areas. Tissue examination was performed by three independent observers with minimal inter-observer variability.

### 4.6. Bioinformatic Analysis

For the analysis of survival, an open source software bioinformatic tool was employed, called the Kaplan–Meier plotter (http://kmplot.com/analysis/index.php?p=background) that includes gene chip and RNA-seq data by GEO, EGA, and TCGA [25].

### 4.7. Statistical Analysis

For statistical analysis the GraphPad Prism Software was employed. Student’s *t*-test for two groups and one-way ANOVA for multiple groups were employed for statistical evaluation. All values were presented as mean values ± standard deviation (STDEV). *p* < 0.05 was regarded as statistically significant.

## 5. Conclusions

In conclusion, the results of the current study demonstrate a unique spatial immune profiling in PDAC patients that features a highly immunosuppressive microenvironment. The existence of dysfunctional T cells with specific spatial distribution in the primary lesion and in metastatic LNs opens a new dimension in tumor-stroma interaction that could refine histopathological evaluation. The latter may facilitate the design of combined therapies with improved efficacy. Along this line the CD47-SIRPα innate immune response checkpoint is a potential promising therapeutic target in PDAC. This might be combined with the T cell-based inhibitor checkpoint therapy, since it can also promote priming of T cell responses by enhancing phagocytosis of cancer cells [47]. Moreover, our data contribute to highlight the complexity of the tumor microenvironment in PDAC and offer a new perspective for more precisely tailored approaches and patient stratification.

## Supplementary Materials

The following are available online at https://www.mdpi.com/2072-6694/12/7/1825/s1, Figure S1: Decreased cytotoxic potential in CD8+ cells in the invasive front (IF) versus the normal pancreatic normal parenchyma adjacent to the tumor (NAT), Figure S2: Negative and positive controls for assaying the probe specificity and the quality of mRNA for the RNAscope analysis, Figure S3: Representative confocal micrographs of RNAscope analysis to assess co-expression of HAVCR2 (green) and/or PDCD1 (red) and CD8A (yellow) in non-malignant pancreatic lesions, Figure S4: Topographical distribution of macrophages along with PD-L1/PD-1 and CD47-SIRPα immune-related druggable axes in pancreatic ductal adenocarcinoma (PDAC) patients, Figure S5: Immune response 2 predominates in PDAC., Figure S6: PD-1 expression in PDAC patients. Representative micrographs of PD-1 immunohistochemical staining in the invasive front (IF), including peritumoral tertiary lymphoid structures (TLS), Figure S7: Topographical expression of CD47 expression and co-expression of CD163/PD-L1 along with CD206-PD-1 in pancreatic ductal adenocarcinoma (PDAC) patients, Table S1: Summary of clinicopathological data, Table S2: Treatment regimen of patients with pancreatic ductal adenocarcinoma (PDAC) receiving neoadjuvant chemotherapy.
