# Supplementary material for: Unique Spatial Immune Profiling in Pancreatic Ductal Adenocarcinoma with Enrichment of Exhausted and Senescent T Cells and Diffused CD47-SIRPα Expression
Source: Cancers (Basel). 2020 Jul 7;12(7):1825. doi: 10.3390/cancers12071825 (PMC7408661; doi:10.3390/cancers12071825)
Supplement: Supplementary file 1 [file cancers-12-01825-s001.zip › supporting information/cancers-828563-supplementary-resubmit.docx]

**Figure S1.** Decreased cytotoxic potential in CD8+ cells in the invasive front (IF) versus the normal pancreatic normal parenchyma adjacent to the tumor (NAT). (**A**) Representative micrographs of the immunohistochemical staining for the CD8, Granzyme A (GZMA) and perforin (PRF1) markers in serial sections. Scale bar: 100μm. Arrows indicate CD8 positive cells in the invasive front (IF). (**B**) Representative micrographs depicting the expression of CD8 (red) and granzyme B (GZMB, green) in the normal parenchyma adjacent to the tumor (NAT). Normal Nuclei were counterstained with DAPI (blue). Arrowheads indicate CD8+ cells, and double arrowheads indicate CD8+GZMB+ cells. Scale bar: 100μm. (**C**) Co-expression of CD8 (emerald) and Granzyme B (GZMB) (brown) assessed by double immunohistochemistry analysis in the normal parenchyma adjacent to the tumor (NAT) and invasive front (IF) from the same patients. Double arrowheads indicate CD8+/GZMB+ double immunopositivity, Single arrowheads indicate GZMB+ immunopositivity and the arrows indicate CD8+ immunostaining. Dashed line depicts IF. Asterisks marks a perineural invasion by a cancerous gland. Scale bar: 200μm.

**Figure S2.** Negative and positive controls for assaying the probe specificity and the quality of mRNA for the RNAscope analysis. Positive controls: *POLR2A* (low, 3-15 copies per cell, green), *PPIB* (medium, 10-30 copies per cell, red) and *UBC* (medium/high, more than 20 copies per cells, yellow). Scale bar: 100μm.

**Figure S3.** Representative confocal micrographs of RNAscope analysis to assess co-expression of *HAVCR2* (green) and/or *PDCD1* (red) and *CD8A* (yellow) in non-malignant pancreatic lesions. The percentage of *CD8A*+ cells expressing *HAVCR2* and/or *PDCD1* is presented in brackets. Cases 3, 8: intraductal papillary mucinous neoplasm (IPMN); Case 7: serous cystadenoma (SC); Case 1: chronic pancreatitis. Double arrowheads depict *CD8A*+ co-expressing *HAVCR2/PDCD1* and single arrowheads depict T lymphocytes expressing only *CD8A*+. Scale bar: 100μm.

**Figure S4.** Topographical distribution of macrophages along with PD-L1/PD-1 and CD47-SIRPα immune-related druggable axes in pancreatic ductal adenocarcinoma (PDAC) patients. (**A**) Heatmap analysis summarising the protein expression profile of immune-related markers in the tumor center (TC) and the invasive front (IF) in PDAC patients without (w/o) or receiving neoadjuvant chemotherapy. Cases received neoadjuvant chemotherapy are numbered in the heat map analysis from left to right as 1-14 (see Table S2). PD-L1(t)]: PD-L1 expression in tumor cells; PD-L1(i) PD-L1 expression in the immune cell compartment. (**B**) Quantification of the cells positive the macrophage markers CD64 and CD206. *p-value<0.05, ** p-value<0.01. Data are expressed as mean +/- STDEV (n=27, patients without neoadjuvant therapy; n=14, patients that received neoadjuvant chemotherapy). (**C**) Representative micrographs of immunohistochemical staining for CD64, CD163 in serial sections of PDAC and a non-malignant case with chronic pancreatitis. Arrowheads indicate the cells positive for the indicated markers. Scale bar: 100μm. (**D**) Heatmap analysis showing the protein expression profile of the macrophage-related markers CD64 and CD206 in non-malignant pancreatic lesions (n=9). #1 and #2: chronic pancreatitis; #3 and #8: intraductal papillary mucinous neoplasm (IPMN); #4: main pancreatic duct lithiasis; #5 and #6: mucinous cystic neoplasm; #7: serous cystadenoma; #9: pseudopapillary neoplasm.

**Figure S5.** Immune response 2 predominates in PDAC. (**A**) RNA scope analysis to examine the level of expression of mRNA specific for IL13 (green), TBX21 (red) and GATA3 (yellow) in the Tumor center (TC) and the invasive front (IF) in PDAC. Representative confocal micrographs. Dashed line depicts the IF. Asterisks show cancer glands. Arrows demonstrate TBX21 positive signal, arrowheads depict GATA3 positive staining and dashed arrows depict IL13 positive signal. Scale bar: 100μm. (**B**) Quantification of mRNA level employing H-score. Data are presented as GATA3/TBX21 ratio. *p-value <0.05 (N = 3). (C) Quantification of mRNA level employing H-score. Data are presented as IL13/TBX21 ratio.

**Figure S6.** PD-1 expression in PDAC patients. Representative micrographs of PD-1 immunohistochemical staining in the invasive front (IF), including peritumoral tertiary lymphoid structures (TLS). Arrows indicate PD-1 positive cells. Asterisks depict peritumoral TLS.

**Figure S7.** Topographical expression of CD47 expression and co-expression of CD163/PD-L1 along with CD206-PD-1 in pancreatic ductal adenocarcinoma (PDAC) patients. (**A**) Reduced CD47 status in PDAC patients that received neoadjuvant chemotherapy. Representative micrographs of CD47 immunohistochemical staining (brown) in the invasive front (IF) and the tumor center (TC) of a patient who did not (w/o) or received neoadjuvant chemotherapy. Dashed line depicts the IF. Scale bar: 100μm. (**B**) Representative micrograph of the double immunofluorescence analysis for CD163 (red) and PD-L1 (green). Nuclei were counterstained with DAPI. Arrowhead indicates CD163 and PD-L1 co-expression. Asterisk denotes cancer gland. Scale bar: 100μm (inset scale bar: 25μm). (C) Representative micrograph of the double immunofluorescence analysis for CD206 (green) and PD-1 (red). Nuclei were counterstained with DAPI. Arrowhead indicates CD206 and PD-1 co-expression. Arrowhead indicates CD163 and PD-L1 co-expression. Asterisk denotes cancer gland. Scale bar: 100μm (inset scale bar: 25μm).
